# Inhibitory effect of novel iron chelator, 1-(N-acetyl-6-aminohexyl)-3-hydroxy-2-methylpyridin-4-one (CM1) and green tea extract on growth of *Plasmodium falciparum*

**DOI:** 10.1186/1475-2875-13-S1-P84

**Published:** 2014-09-22

**Authors:** Somdet Srichairatanakool, Supalerk Thipubol, Wachiraporn Tipsuwan, Chairat Uthaipibull

**Affiliations:** 1 Chiang Mai University, Chiang Mai, Thailand; 2 University of Phayao, Phayao, Thailand; 3 National Science and Technology Development Agency, Pathum Thani, Thailand

## Background

Iron is an essential micronutrient required by all living organisms including malaria parasites (*Plasmodium* spp.) for many biochemical reactions, especially growth and multiplication processes. Therefore, the malaria parasites needs to take up the iron from outside or/and inside the parasitized red blood cells (PRBC). Iron chelators are widely used for treatment of thalassemia-related iron overload and also inhibit parasite growth at levels which are non-toxic to mammalian cells.

## Materials and methods

The inhibitory effect of 1-(N-acetyl-6-amino-hexyl)-3-hydroxypyridin-4-one (CM1) and green tea extract (GTE) on the growth of malaria parasite *P. falciparum* was investigated compared with standard chelators including desferrioxamine (DFO), deferiprone (DFP) and deferasirox (DFX). A flow cytometric technique was used to enumerate the PRBC stained with SYBR Green I fluorescent dye. Labile iron pool (LIP) was assayed using calcein-acetomethoxy (Calcein-AM) fluorescent method.

## Results

The IC_50_ values of DFO, GTE, CM1, DFX and DFP against *P. falciparum* were 14.09, 21.11, 35.14, 44.71 and 58.25 μM, respectively. Importantly, CM1 was more effective in reducing LIP level in the *P. falciparum* culture than DFP (*P* < 0.05).

## Conclusions

CM1 and GTE exhibit anti-malarial activity. They could interfere with uptake of exogenous iron or deplete intracellular labile iron in malaria parasites, leading to inhibition of their growth.

